# OTME-2. Regulation of chromatin accessibility in the hypoxic tumor microenvironment of glioblastoma

**DOI:** 10.1093/noajnl/vdab070.053

**Published:** 2021-07-05

**Authors:** Monika Dzwigonska, Jakub Mieczkowski, Paulina Pilanc, Salwador Cyranowski, Agata Kominek, Katarzyna Piwocka, Bozena Kaminska, Katarzyna B Leszczynska

**Affiliations:** 1 Laboratory of Molecular Neurobiology, Neurobiology Center, Nencki Institute of Experimental Biology, Warsaw, Poland; 2 Gdansk Medical University, International Research Agenda 3P, Gdansk, Poland; 3 Postgraduate School of Molecular Medicine, Medical University of Warsaw, Warsaw, Poland; 4 Laboratory of Cytometry, Nencki Institute of Experimental Biology, Warsaw, Poland

## Abstract

Chromatin structure is often dysregulated in cancers, including glioblastoma (GBM), the most common primary brain tumor in adults. GBM has the poorest prognosis and no efficient cure to date due to diffusive growth into the surrounding brain, preventing complete surgical resection and leading to inevitable tumor relapse. Tumor microenvironment (TME) of GBM contains brain-residing microglia and bone-marrow derived macrophages (collectively known as glioma-associated microglia/macrophages, GAMs) that constitute up to 30% of the tumor mass and promote tumor invasion. Hypoxia (a shortage of oxygen) is a key factor in tumor progression of GBM as it can globally and rapidly alter the gene expression, induce cancer cell invasiveness, stemness and lead to therapy resistance. Hypoxia can enhance the pro-tumorigenic function of GAMs, e.g. by inducing expression of cytokines and cell surface receptors both in GAMs and glioma cells, but little is known about chromatin alterations of GBM under hypoxia. Since regulation of expression of such molecules could depend on the epigenetic alterations, we hypothesize that hypoxia may potently alter the chromatin accessibility and functions of GAMs and glioma cells.

We determine the genome-wide changes in chromatin accessibility in GAMs and glioma cells in response to hypoxic stress using single-cell Pi-ATAC-seq (Protein-indexed Assay of Transposase Accessible Chromatin with sequencing), which allows simultaneous genome-wide assessment of chromatin accessibility and expression of intracellular protein markers in single cells, allowing faithful selection of hypoxic and non-hypoxic cells. Secondly, we are employing an oxygen-dependent co-culture model *in vitro* to study the mechanisms of chromatin alterations in GAMs and glioma cells under controlled hypoxic conditions and test how these changes depend on the glioma - GAMs cross-communication. In summary, we characterize the interactions between innate immune cells and glioma cells by looking at their chromatin alterations under hypoxia.

Supported by the National Science Center grant (Poland) 2019/33/B/NZ1/01556 (KBL).

